# Assessment of the Behavioural Response of Korean Water Deer (*Hydropotes inermis argyropus*) to Different Fence Heights

**DOI:** 10.3390/ani11040938

**Published:** 2021-03-26

**Authors:** Hee-Bok Park, Donggul Woo, Tae Young Choi, Sungwon Hong

**Affiliations:** 1 Research Center for Endangered Species, National Institute of Ecology, Yeongyang-gun 36531, Korea; marinecat80@nie.re.kr; 2 Division of Ecological Conservation, National Institute of Ecology, Seocheon-gun 33657, Korea; martes@nie.re.kr; 3 Department of Animal Science and Biotechnology, Kyungpook National University, Sangju 37224, Korea; 4 Department of Horse, Companion, and Wild Animal Science, Kyungpook National University, Sangju 37224, Korea

**Keywords:** roadkill, fence, non-chemical capture technique, *Hydropotes inermis argyropus*, non-metric multidimensional scaling, generalised linear mixed model

## Abstract

**Simple Summary:**

The present study aimed to investigate the success rate of fences and classify the behavioural responses of Korean water deer (*Hydropotes inermis argyropus*) to different fence heights. The dominant behaviours before the deer crossed the fences by performing vertical and running jumps were recession and rest when the fence heights were lower or higher than 1.2 m, respectively. The general threshold (fence height) for discriminating success or failure was 0.9 m; however, we recommend a fence height of 1.5 m, considering the cost and roadkill risk. Placing exit pathways for deer and eliminating possible resting areas outside fences are essential for reducing the number of successful jump attempts.

**Abstract:**

Fences have been widely implemented to reduce the risk of wildlife–vehicle collisions, wildlife disease spread, and crop damage. To manufacture fences, it is imperative to assess the behavioural responses of the target species. Here, we investigated the success rate of fences and classified eight behavioural responses of Korean water deer (*Hydropotes inermis argyropus*) to different fence heights. We explored the association of 801 behavioural responses and defined a threshold based on 40 events by applying non-metric multidimensional scaling and a binary logistic generalised linear mixed model. With fences lower and higher than 1.2 m, recession and rest were the dominant behaviours, respectively, before the deer crossed the fences by performing vertical and running jumps. Considering all independent events, 0.9 m was the marginal threshold, with highly variable outliers over this value. Placing exit pathways for deer and eliminating possible resting areas outside fences are essential for reducing the number of successful jump attempts. The optimal fence height could differ based on conditional factors; however, we recommend a height of 1.5 m considering the cost and roadkill risk. In conclusion, exploring and classifying the behavioural responses of the target species may be critical for establishing appropriate fence protocols.

## 1. Introduction

Fences have been widely implemented to reduce the risk of wildlife–vehicle collisions, wildlife disease spread, and crop damage [1,2,3,4,5]. Although fences restrain the movement of some endangered species by creating barriers for genetic exchange, building fences can also improve biodiversity by blocking invasive species as well as provide optimal habitats for endangered species to facilitate trophic rewilding and secure populations [6,7,8,9]. However, when erecting fences, it is critical to assess the behavioural responses of the target species [1,10,11,12].

Although there are some studies that address fencing-based mortality reduction of wildlife and deterrence rates at different fence heights, few studies have assessed the behavioural responses of target species to various fence heights [1,2]. According to Clevenger et al. (2001), fencing effectively reduced collisions between vehicles and ungulates (elk (*Cervus elaphus*) and white-tailed deer (*Odocoileus virginianus*)) to 80% [1]. Behavioural responses differ depending on the height, type, and structure of fences and nearby landscapes [12,13,14,15]. In the case of white-tailed deer (*O. virginianus*), deterrence rates were 0%, 14%, 85%, and 100% in response to fences with heights ≤1.5 m, 1.8 m, 2.1 m, and 2.4 m, respectively, within a flat experimental pen with sand soil [2]. High or conspicuously marked fences can obstruct deer when jumping or decrease the frequency of jumping, even though the species can theoretically jump over such fences [2,12]. The location and strength of fences are also relevant for deer when trying to jump [16]. However, although behavioural responses can vary with fence height, specific jumping parameters (e.g., vertical or running jumps and location of the jump) or avoidance behaviours (e.g., recession or rest) have not been elucidated thus far to support an appropriate fence height.

The Korean water deer (*Hydropotes inermis argyropus*) is one of the two subspecies of Chinese water deer [17]. While the population of Chinese subspecies is critically endangered in China, the Korean subspecies is widely distributed throughout South Korea [18,19]. Indeed, in South Korea, the population has grown, leading to socioecological problems, such as crop damage, vehicle collisions, and wildlife disease spread [20,21]. This species is among the most frequent to experience vehicle collisions [22]. To reduce collisions, the Ministry of Environment, together with the Ministry of Land, Infrastructure, and Transport, has built fences along roads and wildlife passages. These fences are generally between 1.2 and 1.5 m in height but flexible according to regional conditions [23,24]. However, few studies have assessed the behavioural responses of water deer to these fences [25].

Specific behavioural responses to fences of different heights could be useful for the implementation of appropriate fences with the goal to reduce collisions [1,26]. For instance, the success rate of vertical and running jumps can vary depending on the fence height. In areas where vertical jumping ability is low, such as in steep areas, the fence height can be decreased. Moreover, the location of jumping and avoidance behaviours may demonstrate how and where to make detours, because the probability of escape is closely related to the behavioural responses of species. Thus, in the present study, we assessed the jumping ability and behavioural responses of water deer to fences of different heights.

## 2. Materials and Methods

### 2.1. Study Area

**Figure 1:**
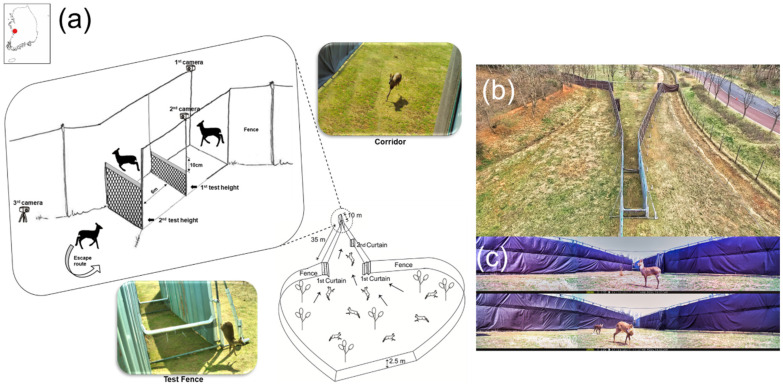
(**a**) Location and diagram of the facility in the deer park. The left upper inset is the enlarged diagram of the test fence area. Two pictures show the water deer in the corridor and test fence. (**b**) Pictures of the capture-jumping test experimental facility (CJEF) and (**c**) water deer in the CJEF.

We investigated the behavioural responses of deer by creating a test facility (Figure 1b) in a rescued deer park (2,223 m^2^, Figure 1a) located at the National Institute of Ecology, Seocheon-gun (36°1′51.90″ N, 126°43′6.26″ E). Three native, wild ungulates, namely water deer, long-tailed goral (*Naemorhedus caudatus*), and Siberian roe deer (*Capreolus pygargus tianschanicus*), inhabit this park under near-wild conditions. The vegetation mostly comprises deciduous trees, such as sawtooth oak (*Quercus acutissima*), sawleaf zelkova (*Zelkova serrata*), and Chinese hackberry (*Celtis sinensis*; https://www.nie.re.kr, accessed on 25 March 2021).

### 2.2. Monitoring of Behavioural Responses to Fences

To assess the jumping ability and behavioural responses of deer to fences, we built a capture-jumping test experimental facility (CJEF; Figure 1a,b) based on the Boma funnel capture system (BCS), as described in previous studies [2,3,27]. The facility had two parts: the BCS and the jumping area (JA). The BCS is often used to drive wild game into a corridor that leads to a truck [27]. To relieve capture stress, we used BCS rather than direct capture and release in the JA. To alleviate stress during prolonged research, we used the corridor of the funnel to design the JA with two consecutive fences perpendicular to the corridor; the first one was 10 cm shorter than the second and often of the height a deer could easily cross (Figure 1c). The distance between the two fences was 6 m. When some deer entered the BCS, we closed the curtains sequentially to funnel them through. The height of the outer wall support was 3 m, and it was covered with oxford fabric (2.5 m × 150 m). We constructed the JA by planting a sheet (plate) measuring 2 m (width) × 10 m (distance) × 3 m (height). We installed polyethylene fences (20 mm × 20 mm) with padding to reduce the risk of injury during the trial. We increased the height by 10 cm each time a deer succeeded in jumping over. If the deer failed to jump, we repeated the test at least six times while maintaining the fence height. To observe the behavioural responses, we implanted two cameras (XR6 Reconyx Inc., Holmen, WI, USA) and one camcorder (HDR-PJ820 Sony Inc., Tokyo, Japan) between the two fences.

After construction, we kept all gates open for 5 days (5–9 April 2017) to allow the deer to adapt to the facility. For 12 days (10–21 April 2017), we monitored 27 deer to evaluate their jumping ability and behavioural responses to the fences. The animal protocol used in this study was reviewed and approved by the ethical committee of the National Institute of Ecology (Table S1).

### 2.3. Classification of Behavioural Responses

We classified the behavioural responses of deer into four major criteria (success, failure, collision, and avoidance). The detailed criteria are listed in Table 1. Success and failure were sub-classified into vertical and running behaviours. When a deer failed, we also considered the location of the failure (fence or side wall). Finally, we also identified avoidance behaviours as either recession or rest.

### 2.4. Statistical Analysis

**Figure 2:**
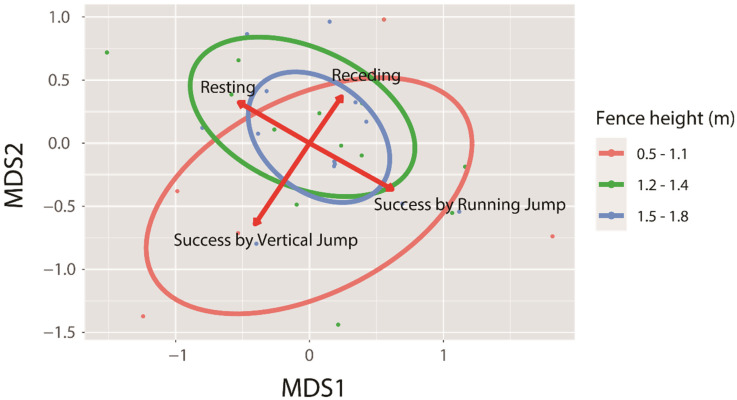
Overall associations among eight behavioural responses (recession, rest, vertical jump to the fence or side wall, running jump to the fence or side wall, and crossing success by a vertical or running jump). Representative responses along the three fence height ranges (0.5–1.1 m, 1.2–1.4 m, and 1.5–1.8 m) are indicated by red arrows, with 95% statistical confidence. Forty events in which deer entered the jumping area (JA) are clustered by height ranges with 95% statistical confidence. The clusters are represented by height ranges and circles of different colours (height: 0.5–1.1 m, red circle; 1.2–1.4 m, green circle; and 1.5–1.8 m, blue circle).

We identified the representative behavioural responses to fence height using cluster analysis (non-metric multidimensional scaling, NMDS) with the vegan package of R 3.2.3 [28,29]. Because we were unable to identify each individual entering the JA, we separately considered all deer as independent individuals. When some individuals concomitantly entered and simultaneously responded to fences (n = 7), we separated these events into independent events, as we could not identify the individuals. We used Bray–Curtis coefficients to ordinate the association of the behavioural response of height in two dimensions using 20 random starts. We used the “stressplot” function of the vegan package to draw a Shepard plot, wherein ordination distances were plotted against event dissimilarities and the fit was shown as a monotone step line of the goodness of fit (*R^2^* = 1 − *S^2^*). To determine the behavioural responses to different fence heights and predict the expected behaviours, we analysed the information on the fence heights using the ggplot2 package [30]. Since the events for some heights only occurred once, general responses could not be determined; therefore, we grouped the fence heights based on the current law regarding fence implementation [23]. The law encourages a fence height of 1.2 or 1.5 m; accordingly, we classified the fence heights into three groups: 0.5–1.1 m, 1.2–1.4 m, and 1.5–1.8 m. The most representative responses to fence heights with a sum of the squares of two axes (>0.1 in the NMDS were identified as significant (*p* < 0.05)) (Figure 2).

To identify general jumping ability, we simply transformed all responses to success (crossing the fence) or failure (not crossing). We applied a binary logistic generalised linear mixed model (GLMM) considering the fence height as the fixed effect and events as the random effect [31,32]. The goodness of model fit was evaluated based on Nagelkerke pseudo *r*^2^ using the companion package of RStudio 1.1.456 [33]. We used an optimal cut-off that equalised specificity (proportion of unoccupied sites correctly predicted) and sensitivity (proportion of occupied sites correctly predicted) and estimated the area under the receiver operating characteristic (ROC) curves using the pROC package of R [34]. The threshold height was obtained by calculating the cut-off [35].

## 3. Results

We collected 801 behavioural responses from 40 events in which the deer entered the JA. Success due to vertical (n = 8, probability (Pr.) = 1.0%) and running (n = 6, Pr. = 0.7%) jumps was very low. When facing a fence of any height, recession was the most frequent behavioural response (n = 519, Pr. = 64.8%), followed by a vertical jump to the sidewall (n = 117, Pr. = 14.6%), failure of a vertical jump to the fence (n = 108, Pr. = 13.5%), a running jump to the fence (n = 19, Pr. = 2.4%), and others (n = 17, Pr. = 2.1%). A running jump to the side wall was the least frequent response (n = 7, Pr. = 0.9%). When the height of the fence increased to 1.2 m, the rate of failure increased substantially. We investigated 11 individuals at a fence height of 1.2 m, but only one could successfully cross over the fence.

While exploring the associations among behavioural responses, we found strong negative associations between rest and success due to a running jump as well as recession and success due to a vertical jump (Figure 2). The association patterns changed with the fence height. Below 1.2 m, recession and success due to a vertical jump were closely related (red circle in Figure 2), whereas over 1.2 m, rest and success due to a running jump were closely related (blue and green circles in Figure 2). As the number of failures due to vertical jumps increased, the frequency of recession increased. Likewise, as the number of failures by running jumps increased, the frequency of resting increased.

**Figure 3:**
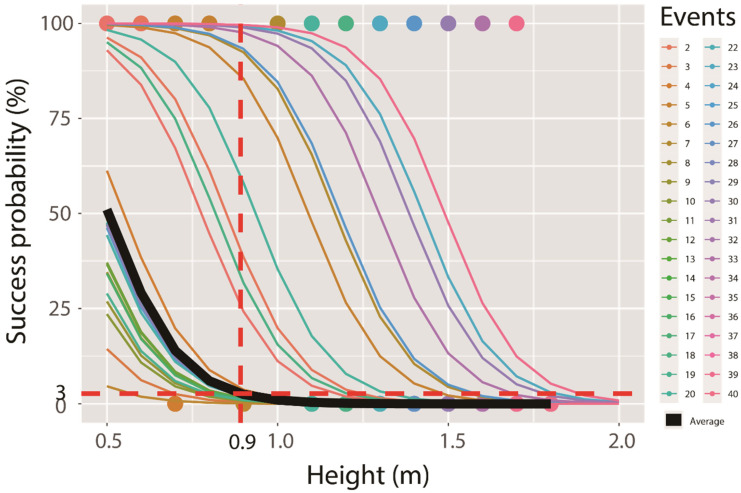
Regression lines according to the binary logistic generalised linear mixed model using fence height (m) from 40 events. The bold black line indicates the marginal relationship, while the thin coloured lines indicate the conditional relationships. The vertical and horizontal dotted lines are for calculating the threshold of fence height at the level of optimal cut-off that equalised specificity and sensitivity.

The results of GLMM suggested that fence height significantly affected the crossing success rate of the deer (coefficient = −9.29 ± 4.61, *p* < 0.05; Nagelkerke *r*^2^ = 0.34, ROC = 0.96). As the height increased to 1 m, the crossing success rate decreased to 0.01%. The general threshold (fence height) for discriminating success or failure was 0.9 m (the point of intersection with the red dotted line), with highly variable outliers above this value (Figure 3).

## 4. Discussion

Our results showed that fence height affected the probability of the examined behavioural responses of Korean water deer (rest or recession vs. success due to vertical or running jumps). Interestingly, there were marked associations among the four responses, depending on the fence height (Figure 2). At a fence height below 1.2 m, recession and success due to a vertical jump were the dominant behavioural responses. Recession seems to be an adverse behaviour to success due to vertical jumps. This suggests that failure due to vertical jumps is strongly and positively associated with the frequency of recession. Thus, the frequency of recession can be reduced by introducing exit pathways. When the deer failed to cross the fence, they would naturally recede; however, if there were an exit, they would not attempt to jump again and simply exit the area. At a fence height above 1.2 m, the deer mostly tried to run and jump to cross it. Upon failure, they rested and retried the jump. Thus, to prohibit jumping, it is important not to place resting areas near fences. With the implementation of additional exits, the deer would be able to exit the fenced areas without resting and avoid attempting to jump again. In summary, by classifying and linking behavioural responses, we can determine the most relevant fence height based on the structure of the nearby landscape.

Our results suggest that some individuals could cross fences with a height above 1.8 m, and in several cases, they could easily jump over the threshold height of 0.9 m; however, even though our model contained uncertainty; based on our results, fences can be implemented flexibly according to the budget and regional conditions. Because our model suggested that a height above 0.9 m may have a greater effect, we should manufacture fences with a height of at least 0.9 m to decrease crossing success. When a larger budget is available or in areas where human activity would not be disturbed, higher fences could be implemented to further reduce variability. As such, higher fences could be implemented in high-risk areas, while fences of minimal height can be implemented in some low-risk areas to reduce costs. Because our model suggests a minimal threshold to achieve the desired effect by the fence, the present evaluation method seems relevant for establishing flexible fence implementation plans [36].

For example, our model suggested a threshold of 0.9 m for fence height, and a fence height of 1 m reduced the crossing success rate to 0.01%; therefore, we recommend the use of fences with a height of at least 0.9 m and, preferably, 1.5 m for Korean water deer. Since the Korean government has recommended the fence height of 1.2 and 1.5 m, all fences are manufactured according to this standard [23,24]. Thus, fences are the most cost-effective at this height. If the required fence height were not 1.2 or 1.5 m, the cost may increase by up to 1.86 times. However, the 30 cm difference between fences of 1.2 and 1.5 m accounts for a small fraction of the fence-building cost (which includes the costs of labour and transport of material to the site). Upon consulting some companies in Korea, we noted a 6% increase in cost per kilometre for building a 1.5 m fence compared with that for building a 1.2 m fence. This small additional cost is offset by the probability that some deer may succeed in jumping the fence under certain circumstances, causing car crashes that may lead to severe injuries to people or even loss of life.

Although we revealed the general behavioural responses of Korean water deer to fences of different heights, there is room to further delineate detailed fence implementation plans. First, non-chemical capture techniques can be modified to improve the accuracy of the building strategy. The CJEF used has the advantage of reducing the capture stress to the target animals, but it was also limiting in that we could not easily identify the individuals that randomly entered the JA. Although we considered all events to be independent to overcome this shortcoming, by identifying individuals, we could better analyse the behavioural responses according to the individuals’ abilities. Thus, the identification of individuals using non-invasive techniques, such as appearance discrimination through camera trapping, is warranted [37]. In addition, behavioural responses may vary according to fence type and season [38] and warrant further research. Finally, the species could also adapt to the circumstances; therefore, further long-term monitoring studies are imperative [39].

## 5. Conclusions

Fences have been widely implemented to reduce the risk of wildlife–vehicle collisions, wildlife disease spread, and crop damage. When constructing fences, the behavioural responses of the target species must be assessed. Here, we investigated and classified the behavioural responses of Korean water deer to fences of different heights by exploring the associations among diverse responses and defining thresholds by applying NMDS and GLMM. For fence heights below and above 1.2 m, recession and rest were the dominant behaviours before crossing the fence, respectively. Considering all independent events, 0.9 m was selected as the marginal threshold height, with highly variable outliers over this value. Creating an exit pathway for deer and eliminating resting areas outside fences may be essential to reduce the number of attempted jumps. The optimal fence height may differ based on conditional factors; however, we recommend a height of 1.5 m considering the fence cost and roadkill risk.

Although this study was species-specific and spatially limited, the methods used could apply to a wider range of species and areas that require effective fencing solutions. The novelty of the methods applied here relate to two elements: (i) non-chemical capture systems have never (to our knowledge) been adapted to drive animals safely into the corridor to investigate their jumping ability, and (ii) the method of calculating the fence-height threshold and measuring the behavioural responses of target species to the fences can be applied to other species for which effective fencing options are needed. Although the non-chemical capture system and statistical methods used here could be developed further, the approach to exploring and classifying the behavioural responses of wild animals used in these experiments is relevant for establishing appropriate wildlife fencing.

## Figures and Tables

**Table 1:** Classification and description of response behaviours.

Major Behaviour	Related Behaviour	Location	Detailed Description
Success	Vertical jump	-	Success due to a vertical jump
	Running jump	-	Success due to a running jump
Failure	Vertical jump	Fence	Failure due to a vertical jump to the fence
		Wall	Failure due to a vertical jump to the side wall
	Running jump	Fence	Failure due to a running jump to the fence
		Wall	Failure due to a running jump to the side wall
Collision	-	-	Collision with the fence or side wall without a jump
Avoidance	Recession	-	Recession
	Rest	-	Rest by either standing or sitting

## Data Availability

Data available on request due to restrictions.

## Supplementary Materials

The following are available online at https://www.mdpi.com/article/10.3390/ani11040938/s1, Table S1. Notes regarding handling experimental ethical issues.
