# Supplementary material for: Assessment of the Behavioural Response of Korean Water Deer (Hydropotes inermis argyropus) to Different Fence Heights
Source: Animals (Basel). 2021 Mar 26;11(4):938. doi: 10.3390/ani11040938 (PMC8067234; doi:10.3390/ani11040938)
Supplement: Supplementary file 1 [file animals-11-00938-s001.pdf]

Table S1. Notes regarding the handling of experimental ethical issues

Ethical issues	Application
Capturing may cause severe stress to the individuals	To relieve stress, we adapted a non-chemical technique—the Boma funnel capture system (BCS). BCS is often used for driving wild game into a corridor, which leads to a truck. When some deer entered the system, we closed the curtains sequentially to funnel them through. This system does not require animal capture, and can thus substantially relieve the stress.
Prolonged research duration may aggravate the animals	1. To alleviate stress during prolonged research duration, we set up two consecutive fences perpendicular to the corridor. At a height lower than 1 m, which the water deer can easily cross, we used two fences to reduce the research time.<br>2. When the fence height increased and the water deer did not try to jump, we drove them by making a sound to reduce research time.<br>3. When the deer crossed the fence, we stopped inspecting their ability at that height.
During jumping, the deer may be injured	To reduce the risk of injury, we installed polyethylene fences with padding.
